# The Impact of Blood Transfusion on Recurrence and Mortality Following Colorectal Cancer Resection: A Propensity Score Analysis of 4,030 Patients

**DOI:** 10.1038/s41598-018-31662-5

**Published:** 2018-09-06

**Authors:** Hsiang-Ling Wu, Ying-Hsuan Tai, Shih-Pin Lin, Min-Ya Chan, Hsiu-Hsi Chen, Kuang-Yi Chang

**Affiliations:** 1 Department of Anesthesiology, Taipei Veterans General Hospital, Taipei, Taiwan; 2 Department of Surgery, Taipei Veterans General Hospital, Yuli Branch, Hualien Taiwan; 3 School of Medicine, National Yang-Ming University, Taipei, Taiwan; 4 Department of Anesthesiology, Shuang Ho Hospital, Taipei Medical University, New Taipei City, Taiwan; 5 Department of Anesthesiology, School of Medicine, College of Medicine, Taipei Medical University, Taipei, Taiwan; 6 Department of Technology Application and Human Resource Development, National Taiwan Normal University, Taipei, Taiwan; 7 Division of Biostatistics, Graduate Institute of Epidemiology and Preventive Medicine, College of Public Health, National Taiwan University, Taipei, Taiwan

## Abstract

Whether blood transfusion exacerbates cancer outcomes after surgery in humans remains inconclusive. We utilized a large cohort to investigate the effect of perioperative blood transfusion on cancer prognosis following colorectal cancer (CRC) resection. Patients with stage I through III CRC undergoing tumour resection at a tertiary medical center between 2005 and 2014 were identified and evaluated through August 2016. Propensity score matching was used to cancel out imbalances in patient characteristics. Postoperative disease-free survival (DFS) and overall survival (OS) were analysed using Cox regression model. A total of 4,030 and 972 patients were analysed before and after propensity score matching. Cox regression analyses demonstrated blood transfusion associated with shorter DFS and OS before and after matching (hazard ratio: 1.41, 95% CI: 1.2–1.66 for DFS; 1.97, 95% CI: 1.6–2.43 for OS). Larger transfusion volume was linked to higher overall mortality (≤4 units vs. nil, HR = 1.58; >4 units vs. nil, HR = 2.32) but not more cancer recurrence. Preoperative anemia was not associated with decreased survival after adjusting covariates. Perioperative blood transfusion was associated with worse cancer prognosis after curative colorectal resection, independently of anemia status. Strategies aimed at minimizing transfusion requirements should be further developed.

## Introduction

Colorectal cancer (CRC) is one of the most prevalent malignancies worldwide, with an increasing incidence reported in many countries^1^. A sizable portion of patients with CRC have perioperative anemia, which may result from malnutrition, adjuvant or neoadjuvant chemotherapy, systemic inflammation, spontaneous tumour bleeding, or surgical blood loss^2^. Anemia, the major determinant for perioperative blood transfusions, has been associated with impaired survival in patients for cancer surgery^3,4^.

While blood transfusion is mandatory to correct the physiologic abnormalities associated with anemia in many circumstances, it has long been postulated to exert detrimental effects on the immune function of patients through transfusion-associated immunomodulation^5^. In a rat model, a link between progressing malignancy and aged erythrocytes was reported^6^. However, in humans, whether perioperative blood transfusions negatively influence cancer outcomes remains an issue of great debate, with augmented risks reported in some studies^7–9^ but not in others^10–12^. Major shortcomings in previous studies have been frequent small sample size (<1,000 patients in all but three analyses^8,9,12^), inconsistent and incomplete consideration of confounders. Almost all of the patients in the 3 largest studies were recruited during the 1970s through 1990s, and as such these analyses were limited in their abilities to reflect recent advances in cancer treatment. Despite several existing meta-analyses, drawing inferences across studies is difficult because of sample heterogeneity and different study designs^13,14^.

Given the limitations of performing a randomized trial with allogeneic blood transfusion, we conducted a retrospective cohort study containing a large number of patients carefully controlled for important prognostic variables and evaluated meticulously for cancer outcomes at a tertiary medical center. To evaluate the putative impact of perioperative blood transfusions, postoperative disease-free and overall survival of patients was analysed with Cox regression models and propensity-scoring methods to adjust potential imbalances in baseline characteristics. Comprehensive predictors for oncologic outcomes of CRC were taken into account in the analysis to minimize potential confounding effects.

## Results

### Characteristics of the Patients

The median follow-up time of the 4,030 patients was 46.1 months with interquartile range from 24.7 to 73.1 months. The transfusion group was more likely to be older and have comorbidities (diabetes, coronary arterial disease, etc), American Society of Anesthesiologists (ASA) class ≥3, higher pretreatment carcinoembryonic antigen (CEA) level, platelet count, international normalized ratio (INR) value, and lower hemoglobin concentration. Besides, patients receiving perioperative transfusion were more likely to have right-sided tumour and longer anaesthesia time (Table 1). After propensity score matching, the final sample of 486 matched pairs of patients was analysed, and no more unbalanced variable was found between the two groups (Table 1). Table 2 shows the details of cancer staging and pathologic features of the two groups. The transfusion subjects had more advanced cancer stage and were more likely to have tumours of poor differentiation, mucinous histology, and lymphovascular invasion. Note that all the pathologic imbalances are compensated after matching.

**Table 1:** Patient demographics.

	Before matching			After matching		
	Transfusion (N = 1,010)	Non-transfusion (N = 3,020)	Standardized difference	Transfusion (N = 486)	Non-transfusion (N = 486)	Standardized difference
Age, year	73 ± 12	66 ± 13	50.6	72 ± 12	73 ± 12	6.7
Gender, male	632 (62.6%)	1847 (61.2%)	2.9	299 (61.5%)	327 (67.3%)	12.1
ASA class ≥3	572 (56.6%)	800 (26.5%)	64.2	251 (51.6%)	272 (56%)	8.7
Comorbidites						
Diabetes	304 (30.1%)	568 (18.8%)	26.5	251 (51.6%)	272 (56.0%)	4.1
Coronary artery disease	179 (17.7%)	284 (9.4%)	24.5	130 (26.7%)	139 (28.6%)	2.7
Heart failure	118 (11.7%)	141 (4.7%)	25.8	80 (16.5%)	85 (17.5%)	6.0
Stroke	102 (10.1%)	140 (4.6%)	21.0	48 (9.9%)	57 (11.7%)	3.5
Chronic kidney disease	262 (25.9%)	305 (10.1%)	42.1	44 (9.1%)	49 (10.1%)	9.4
Pretreatment CEA, μg·L^−1^	3.45 (2.29–8.89)	2.60 (1.94–4.41)	38.5	3.16 (2.22–6.54)	3.3 (2.23–7.7)	5.4
Hemoglobin conc., g·dL^−1^	10.1 ± 1.8	12.8 ± 1.6	156.5	11.1 ± 1.6	11.1 ± 1.5	1.7
Platelet count, 10^3^·μL^−1^	287 ± 122	236 ± 77	49.7	258 ± 104	268 ± 108	9.4
INR > 1	506 (50.3%)	1380 (45.8%)	9.2	222 (45.7%)	229 (47.1%)	9.4
Tumour location, left-sided	573 (56.7%)	2314 (76.6%)	43.2	325 (66.9%)	320 (65.8%)	2.2
Epidural block	182 (18.0%)	504 (16.7%)	3.5	87 (17.9%)	106 (21.8%)	9.8
Anaesthesia time, min	300 (240–375)	285 (240–345)	31	300 (240–375)	315 (270–390)	17.7
Laparoscopic surgery	40 (4%)	261 (8.6%)	19.4	30 (5.2%)	13 (2.3%)	15.7
Preoperative C/T ± R/T	259 (8.6%)	93 (11.6%)	3.1	50 (10.3%)	58 (11.9%)	5.2
Postoperative C/T (<90 days)	1334 (44.2%)	364 (45.4%)	8.3	225 (46.3%)	229 (47.1%)	1.6
Postoperative R/T (<90 days)	38 (1.3%)	8 (1.0%)	7.1	12 (2.5%)	10 (2.1%)	2.8

Values were mean ± SD, counts (percent), or median (interquartile range). Continuous variables are analysed with Wilcoxon rank-sum tests; categorical variables are analysed with Pearson chi-square tests or Mann-Whitney U tests, as appropriate. Standardized difference is the difference in mean, proportion or rank divided by the pooled standard error, expressed as percentage; imbalance is defined as absolute value greater than 20 (small effect size). ASA: American Society of Anesthesiologists; CEA: carcinoembryonic antigen; Con.: concentration; INR: international normalized ratio; C/T: chemotherapy; R/T: radiotherapy.

**Table 2:** Cancer staging and pathologic features.

	Before matching			After matching		
	Transfusion (N = 1,010)	Non-transfusion (N = 3,020)	Standardized difference	Transfusion (N = 573)	Non-transfusion (N = 573)	Standardized difference
AJCC stage			27.3			**0.2**
Stage I	231 (26.3%)	847 (28.0%)		84 (17.3%)	82 (16.9%)	
Stage II	351 (40.0%)	1158 (38.3%)		208 (42.8%)	212 (43.6%)	
Stage III	295 (33.6%)	1015 (33.6%)		194 (39.9%)	192 (39.5%)	
**Pathologic features**						
Tumour differentiation			10.8			**1.3**
Good	44 (4.4%)	248 (8.3%)		28 (5.8%)	22 (4.5%)	
Moderate	879 (87.6%)	2573 (86.4%)		423 (87.0%)	431 (88.7%)	
Poor	80 (8.0%)	157 (5.3%)		35 (7.2%)	33 (6.8%)	
Mucinous histology	51 (5.1%)	103 (3.5%)	8.1	26 (5.3%)	22 (4.5%)	**3.8**
Signet-ring histology	29 (2.9%)	73 (2.5%)	2.8	11 (2.3%)	12 (2.5%)	**1.4**
Lymphovascular invasion	210 (20.9%)	497 (16.7%)	10.9	88 (18.1%)	94 (19.3%)	**3.2**
Perineural invasion	89 (8.9%)	210 (7.1%)	6.8	43 (8.8%)	40 (8.2%)	**2.2**

Values were mean ± SD, counts (percent), or median (interquartile range). Continuous variables are analysed with Wilcoxon rank-sum tests; categorical variables are analysed with Pearson chi-square tests or Mann-Whitney U tests, as appropriate. AJCC: American Joint Committee on Cancer.

### Disease-Free Survival

The 3-yr and 5-yr disease-free survival rates were 71.4% (95% CI: 68.3–74.5%) and 66.7% (95% CI: 63.2–70.2%) in the transfusion group and 83.5% (95% CI: 82.1–84.9%) and 80.3% (95% CI: 78.7–81.9%) in the non-transfusion group, respectively. The univariate analysis revealed several significant risk factors for cancer recurrence (Table 3), including blood transfusion, lower hemoglobin concentration, ASA class ≥3, chronic kidney disease, higher pretreatment CEA level, longer anaesthesia time, advanced cancer stage, specific pathologic findings (poor differentiation, mucinous or signet-ring histology, lymphovascular invasion, and perineural invasion), preoperative chemotherapy and/or radiotherapy, and postoperative chemotherapy or radiotherapy.

**Table 3:** Univariate analysis of cancer recurrence and all-cause mortality before matching.

	Cancer recurrence			All-cause mortality		
	HR	95% C.I.	*p*	HR	95% C.I.	*p*
Blood transfusion	1.86	1.61–2.16	<0.001	3.21	2.67–3.87	<0.001
Hemoglobin concentration	0.93	0.9–0.96	<0.001	0.82	0.79–0.86	<0.001
Platelet count	1.00	1–1	0.008	1	1–1	0.707
INR (>1 vs. ≤1)	1.14	0.99–1.31	0.071	1.37	1.13–1.66	0.001
Age	1	1–1.01	0.183	1.05	1.04–1.05	<0.001
Gender (M vs. F)	1.08	1.00–1.16	0.039	1.2	1.09–1.33	<0.001
ASA class ≥ 3	1.34	1.16–1.54	<0.001	2.92	2.42–3.52	<0.001
Diabetes	1.14	0.97–1.34	0.123	1.65	1.35–2.02	<0.001
Coronary arterial disease	1	0.8–1.25	0.994	1.88	1.49–2.37	<0.001
Heart failure	0.98	0.73–1.32	0.904	2.72	2.08–3.58	<0.001
Stroke	1.23	0.92–1.64	0.159	2.08	1.51–2.85	<0.001
Chronic kidney disease	1.26	1.04–1.52	0.020	2.29	1.85–2.83	<0.001
Pretreatment CEA*	2.58	2.27–2.92	<0.001	2.1	1.75–2.52	<0.001
Epidural block	0.85	0.71–1.03	0.099	0.89	0.71–1.12	0.327
Anaesthesia time**	1.57	1.33–1.86	<0.001	1.59	1.27–1.98	<0.001
Laparoscopic surgery	0.87	0.65–1.15	0.323	0.67	0.43–1.05	0.080
Preoperative C/T ± R/T	2.06	1.69–2.51	<0.001	1.59	1.2–2.12	0.001
Postoperative C/T	2.77	2.38–3.22	<0.001	1.35	1.12–1.62	0.002
Postoperative R/T	3.29	2.27–4.77	<0.001	2.63	1.57–4.4	<0.001
Right- vs. left-sided tumour	0.88	0.75–1.03	0.109	1.22	1–1.49	0.050
AJCC Stage			<0.001			<0.001
Stage II vs. I	3.58	2.6–4.91	<0.001	1.79	1.32–2.44	<0.001
Stage III vs. I	8.75	6.45–11.87	<0.001	3.09	2.3–4.15	<0.001
Tumour differentiation			<0.001			0.002
Moderate vs. good	2.42	1.64–3.58	<0.001	2.18	1.34–3.54	0.002
Poor vs. good	4.46	2.85–6.98	<0.001	2.9	1.59–5.27	0.001
Mucinous histology	1.57	1.16–2.14	0.004	1.77	1.22–2.58	0.003
Signet-ring histology	2.27	1.63–3.16	<0.001	1.46	0.84–2.53	0.183
Lymphovascular invasion	2.57	2.21–2.99	<0.001	2.09	1.69–2.58	<0.001
Perineural invasion	3.16	2.61–3.82	<0.001	2.46	1.86–3.26	<0.001

HR: hazard ratio; INR: international normalized ratio; M: male, F: female; ASA: American Society of Anesthesiologists; CEA: carcinoembryonic antigen; C/T: chemotherapy; R/T: radiotherapy; AJCC: American Joint Committee on Cancer.

*On base-10 logarithmic scale; **On base-2 logarithmic scale.

After the model selection, patients with blood transfusion have higher adjusted risk of cancer recurrence (HR = 1.41, 95% CI = 1.2–1.66; *p* < 0.001). The association was independent of pre-surgery anemia status (hemoglobin concentration <or≥ 10.0 g·dL^−1^). (Supplementary Table S2) Other independent prognostic factors for disease-free survival included preoperative CEA level (on base-10 logarithmic scale, HR = 1.84), cancer stage (II vs. I, HR = 2.83; III vs. I, HR = 5.82), anaesthesia time (on base-2 logarithmic scale, HR = 1.28), pathologic lymphovascular invasion (HR = 1.37) and perineural invasion (HR = 1.73), signet-ring histology (HR = 1.6), preoperative chemotherapy and/or radiotherapy (HR = 2.19), and postoperative radiotherapy (HR = 2.22) (Table 4). With respect to the propensity score related analyses, both the covariate-adjusted (HR: 1.47, 95% CI: 1.18–1.83, *p* = 0.001) and quintile-stratified propensity score analyses (pooled HR: 1.51, 95% CI: 1.23–1.86, *p* < 0.001) demonstrated significant association between transfusion and cancer recurrence after surgery. After the propensity score matching, stratified Cox regression analysis also shows significantly increased risk of cancer recurrence in the transfusion group (HR = 1.38, 95% CI = 1.02–1.87; *p* = 0.035) (Table 4). Note that no significant dose-response relation was found between the volume of perioperative transfusion and risk of cancer recurrence (≤4 units vs. nil, adjusted HR: 1.39, *p* = 0.001; >4 units vs. nil, adjusted HR = 1.43, *p* < 0.001; >4 units vs. ≤4 units, adjusted HR = 1.03, *p* = 0.81) (Supplementary Table S3).

**Table 4:** Forward model selection for disease-free and overall survival before matching.

	HR	95% C.I.	*p*
Disease-free survival			
Blood transfusion	1.41	1.20–1.66	<0.001
Pretreatment CEA*	1.84	1.60–2.10	<0.001
Anaesthesia time**	1.28	1.06–1.53	0.010
Stage			<0.001
II vs. I	2.83	2.03–3.95	<0.001
III vs. I	5.82	4.20–8.07	<0.001
Lymphovascular invasion	1.37	1.14–1.64	0.001
Perineural invasion	1.73	1.4–2.15	<0.001
Signet-ring histology	1.6	1.13–2.26	0.009
Preoperative C/T ± R/T	2.19	1.77–2.71	<0.001
Postoperative R/T	2.22	1.50–3.29	<0.001
Overall survival			
Blood transfusion	1.97	1.6–2.43	<0.001
Age	1.03	1.02–1.04	<0.001
Gender (M vs. F)	1.25	1.01–1.54	0.042
ASA class ≥ 3	1.62	1.30–2.04	<0.001
Heart failure	1.42	1.05–1.92	0.023
Chronic kidney disease	1.48	1.17–1.88	0.001
Pretreatment CEA*	1.57	1.29–1.92	<0.001
Stage			<0.001
II vs. I	1.3	0.93–1.8	0.122
III vs. I	2.19	1.58–3.04	<0.001
Lymphovascular invasion	1.41	1.1–1.81	0.007
Perineural invasion	1.51	1.10–2.07	0.010
Preoperative C/T ± R/T	2.18	1.60–2.96	<0.001

HR: hazard ratio; CEA: carcinoembryonic antigen; C/T: chemotherapy; R/T: radiotherapy; INR: international normalized ratio; ASA: American Society of Anesthesiologists.

*On base-10 logarithmic scale; **On base-2 logarithmic scale.

### Overall Survival

The 3-yr and 5-yr overall survival rates were 83.4% (95% CI: 80.9–85.9%) and 74.4% (95% CI: 70.9–77.9%) in the transfusion group and 95.2% (95% CI: 94.4–96%) and 91.5% (95% CI: 90.3–92.7%) in the non-transfusion group, respectively. In the univariate analysis, variables associated with shorter survival were perioperative blood transfusion, lower hemoglobin concentration, higher INR value, older age, male, ASA class ≥3, comorbidities (diabetes, coronary arterial disease, etc), higher pretreatment CEA level, longer anaesthesia time, advanced cancer stage, specific pathologic findings (poor differentiation, mucinous histology, lymphovascular invasion, and perineural invasion), preoperative chemotherapy and/or radiotherapy, and postoperative chemotherapy or radiotherapy (Table 3).

After adjusting covariates, patients with blood transfusion have higher risk of overall mortality (HR = 1.97, 95% CI = 1.6–2.43; *p* < 0.001 by log-rank test). After the model selection, patients with blood transfusion have higher adjusted risk of all-cause mortality (HR = 1.97, 95% CI = 1.6–2.43; *p* < 0.001). Also, the association was independent of pre-surgery anemia status (hemoglobin concentration < or ≥10.0 g·dL^−1^). (Supplementary Table S2) Multivariable analysis identified other prognostic determinants for overall survival (Table 4), including older age (HR = 1.03), male (HR = 1.25), ASA class ≥3 (HR = 1.62), heart failure (HR = 1.42), chronic kidney disease (HR = 1.48), higher pretreatment CEA (on base-10 logarithmic scale, HR = 1.57), cancer stage (III vs. I, HR = 2.19), pathologic lymphovascular invasion (HR = 1.41), perineural invasion (HR = 1.51), and preoperative chemotherapy and/or radiotherapy (HR = 2.18). Furthermore, both the covariate-adjusted (HR: 1.84, 95% CI: 1.38–2.45, *p* < 0.001) and quintile-stratified (pooled HR: 1.95, 95% CI: 1.5–2.53, *p* < 0.001) propensity score analytical methods obtained similar results to the multivariable regression analysis. After the propensity score matching, perioperative blood transfusion remained a significant risk factor of mortality (HR = 2.00, 95% CI = 1.27–3.15; *p* = 0.003) (Table 4). Notice that there existed a significant dose-response relation between transfusion volumes and risk of overall mortality (≤4 units vs. nil, HR = 1.58, *p* = 0.001; >4 units vs. nil, HR = 2.32, *p* < 0.001; >4 units vs. ≤4 units, adjusted HR = 1.46, *p* = 0.012) (Supplementary Table S3).

## Discussion

Whether perioperative blood transfusions have a deleterious effect on cancer recurrence or survival remains a controversial issue. The circumstances under which patients receive blood products are likely to influence cancer prognosis. To evaluate whether increased incidence of tumour recurrence is causally related to the blood transfusions, one should consider various important confounding factors, including preoperative functional status, the presence of preoperative anemia, tumour stage and type, duration and type of anaesthesia, etc^5^. The substantial weaknesses of previous reports are small sample size and heterogeneous patients, which made it difficult to assess the risk of blood transfusion.

Our results showed that patient characteristics were strongly biased regarding blood transfusions. Patients requiring blood transfusions had older age, more comorbidities, more aggressive cancer, and complicated clinical courses. However, after optimal adjustment for these imbalances, the link between blood transfusions and worse cancer prognosis remained significant in propensity score-adjusted analyses. Multivariable Cox regression models yielded similar results after considering critical clinicopathologic predictors. In our study the risk of blood transfusion on recurrence and mortality are compatible with the results of prior meta-analyses^13,14^.

Importantly, the previous studies did not clarify the impact of transfusions independent of anemia status with regard to clinical outcomes in colorectal cancer patients. Transfusions could be a proxy of aggressive tumours, causing severe anemia and higher risk of recurrence, instead of a direct cause of recurring by themselves. In our analysis, the Cox regression models, including variables such as transfusions, preoperative anemia, cancer stages, etc, showed it was not anemia but transfusions that actually correlated with worsening cancer outcomes. Furthermore, the results were virtually unaffected when analysing transfusion stratified by preoperative anemia status; i.e. the latter had no obvious effect modification on the former in terms of colorectal cancer prognosis. These results suggested that transfusion and its contributors during the intraoperative (e.g. extent of resection, surgical blood loss, operative techniques) and postoperative periods (e.g. complications) played a crucial role in cancer control.

Anemia has been reported as an independent risk factor for adverse events of myocardial infarction, stroke, death within 30 days of operation, and an increased hospital length of stay following colorectal surgery^15^. However, there are relatively few studies related to preoperative anemia and the risk for cancer recurrence and death in patients operated for colorectal cancer^3,16^. Our results showed preoperative anemia was not linked to increased risk of recurring or death after adjusting covariates, which is discordant with prior investigations^3,4,16^. Recent evidence suggested that surgical patients are able to tolerate lower hemoglobin levels than was previously believed, even in the critically ill. The FOCUS trial indicated restrictive transfusion strategies (a hemoglobin threshold of 7–8 g·dL^−1^) did not increase in-hospital morbidity, short-term or long-term mortality rates compared with liberal strategies (a hemoglobin level of 9–10 g·dL^−1^) in patients undergoing surgery for hip fracture^8,17^. A large meta-analysis showed implementing restrictive transfusion strategies may reduce the incidence of health care-associated infections^18^. Of note, although many studies have been conducted with varying patient conditions, there are few randomized trials focused on the hemoglobin threshold for blood transfusion in oncologic surgery.

The meta-analysis by Amato and Pescatori demonstrated that the risk of cancer recurrence elevated by 40, 69, and 102% after 1 to 2, 3 to 4, and >5 units of packed RBC transfusion, respectively, although significant heterogeneity was detected in the analysis. Our results showed patients with larger volume of perioperative transfusions were merely associated with a greater chance of mortality but not recurrence. It is unclear whether there exists a threshold dose of blood products to produce tumour-promoting effects. We speculate that the impact of transfusion volume may be related to the complexity of operation and cancer aggressiveness, reflecting perioperative course and disease severity rather than the amount of blood transfusions per se^19^. Furthermore, competing causes of death may be another explanation for the dose-response association between blood transfusion and overall mortality rather than cancer recurrence.

It is hypothesized that the detrimental effect of allogenic blood transfusion on cancer outcomes results from immunological derangements caused by transfused leucocytes, including changes in circulating lymphocytes, helper T-cell, suppressor T-cell ratios, and B-cell function^2^. Moreover, one prospective cohort study demonstrated that patients on chronic immunosuppressive therapy have significantly worse long-term oncologic outcomes^20^. However, randomized controlled trials demonstrated that autologous or leukocyte-depleted blood transfusions does not improve oncologic outcomes in patients with colorectal cancer compared with allogeneic transfusions^7,21^. Besides, a recent study reported that transfusion reduction initiative did not prolong disease-free survival in colorectal cancer patients^22^. There are ethical concerns in performing a trial concerning blood transfusion versus no blood transfusion. For the clinician, it is important to optimize the patient medically before surgery, minimize perioperative blood loss, and reduce transfusion requirements.

Several limitations are inherent in this study’s retrospective and observational design. First, the patients were not randomized and clinical care was not standardized and the effects of unmeasured confounding variables cannot be further evaluated. However, for ethical reasons, it is difficult to perform a controlled trial that will ascertain an independent effect of allogeneic blood transfusion on cancer recurrence. A large cohort study applying propensity score matching may be one of the best alternative study designs. Second, we did not further assess the effect of surgical blood loss, other blood products (e.g. platelet concentrates and fresh-frozen plasma) and operative variables by reason of data availability that might alter immune responses and affect oncologic outcomes after surgery^5^.

Third, other variables relevant to platelet concentration and INR (e.g. hepatitis profile, liver cirrhosis, white blood cell count, neutrophil/lymphocyte ratio, platelet/lymphocyte ratio) were not included in the analysis due to data unavailability. Fourth, this study was conducted in a single medical center and the external validity of the results awaits more investigation.

In conclusion, perioperative blood transfusion was significantly associated with increased cancer recurrence and overall mortality in patients after curative colorectal cancer resection, independently of preoperative anemia status. Our findings provided more insights into elucidating the associations among blood transfusion, anemia and postoperative oncologic outcomes in colorectal cancer surgery. Well-designed prospective studies are suggested to explore the casual relationship between allogeneic blood transfusion and decreased survival in patients for cancer surgery.

## Methods

The present study was approved by the Institutional Review Board of Taipei Veterans General Hospital (IRB-TPEVGH No. 2015-11-010CC) and based on the databank of the authors’ institution, one of the largest tertiary medical centers in northern Taiwan. All research was performed in accordance with relevant guidelines and regulations. The written informed consent was waived by the Institutional Review Board, and the whole datasets were anonymized and de-identified before analysis.

### Setting and Patient Selection

After a review of medical records in the electronic medical database, 5741 patients undergoing primary resection for histologically proven colorectal cancer between January 2005 and December 2014 were identified, and 350 patients were excluded due to missing data about demographics, blood product transfusion, or clinicopathologic predictors. Also, 999 patients with distant metastases at the time of operation, 166 patients with pathology-proven carcinoma *in-situ*, and 46 patients with non-adenocarcinoma were excluded. Finally, 150 patients were excluded due to follow-up interval less than 30 days. A total of 4,030 patients were selected for further analyses after the exclusion processes. Patients were divided in two groups: patients who did and their counterparts who did not receive perioperative blood transfusions. Perioperative transfusion was defined as any allogeneic red blood cell (RBC) transfusions given within 7 days of surgery.

### Data Collection

We utilized the medical database to determine the baseline characteristics and risk factors for cancer recurrence and mortality, including demographics, pre-treatment CEA level^23^, and pathologic findings (tumour differentiation^24^, mucinous or signet-ring histology^25^, lymphovascular invasion^26^, and perineural invasion^27^); whether preoperative or postoperative adjuvant chemotherapy or radiotherapy was used. Tumour nodes metastasis (TNM) staging was translated into stages I to III according to the American Joint Committee on Cancer criteria (AJCC-7 staging system)^28^. Tumour locations were divided into right-sided (cecum to splenic flexure) and left-sided tumours (splenic flexure to rectum). In addition, the measurements of laboratory tests prior to surgery were also retrieved, including hemoglobin^29^ and platelet concentration and values of INR. The current status of each patient was based on the documentation of follow-up visits to the hospital’s outpatient clinics or subsequent admissions. All the data were extracted by specialist anaesthesiologists who were not involved in data analysis. The quality of the dataset was verified through random sampling by the authors.

### Follow-up and Criteria for Recurrent Cancer

Patients with node-positive disease routinely received adjuvant chemotherapy (in the form of leucovori and oxaliplatin or 5-fluorouracil; capecitabine; tegafur-uracil) or radiotherapy according to current treatment guidelines, and was defined as any therapy given within 90 days of surgery. Standard surveillance was regularly performed after resection surgery if the patient would be eligible for curative-intent surgery, including CEA tests every 3 to 6 months for 2 years, abdomen and chest computed tomography (CT) scans every 6 to 12 months for at least 3 years, pelvis CT every 3 to 6 months for 2 years for rectal cancer. Characteristic abnormalities detected by imaging studies (CT, magnetic resonance imaging, sonography, bone scan, or plain film) were accepted as evidence of metastatic or locoregional recurrence. If possible, the presence of recurrent cancer was confirmed by histological examinations. Data were collected up to the end of August 2016.

The primary endpoint was disease-free survival, which was defined as time from the date of surgery to the date of cancer recurrence. The secondary endpoint was overall survival, defined as time from the date of surgery to the date of death. For those without the event of cancer recurrence or death, their survival times are regarded as the corresponding censored observations.

### Data Analysis and Statistics

Patient characteristics, surgical data and pathologic findings were compared between groups using t tests, Mann-Whitney U tests and chi square tests as appropriate. Patients without recurrence or alive were censored in the corresponding survival analyses at the last observed day before the end of follow-up time (August 31, 2016). A two-sided significance level of 0.05 was used to assess statistically significant difference. Cox proportional hazards regression model was used to compare the risks of cancer recurrence and overall mortality between groups and to evaluate influences of other collected variables on both outcomes in the univariate analysis. Multivariable models were applied to adjust other independent risk factors obtained from the forward model selection processes with an entry criterion of 0.05. Only complete cases (93.2%) without missing values in the collected variables were analyzed in the multivariable analysis. To account for the potential imbalances in baseline characteristics and pathologic findings, logistic regression analysis was implemented to create propensity scores by incorporating collected variables in the model and the analytical results are presented in Supplementary Table S1^30^. Three propensity score methods were applied to the evaluation of transfusion effects on cancer recurrence and overall survival. First, the obtained propensity scores were directly used as a covariate to adjust for the transfusion effect on cancer recurrence or overall survival in the Cox regression analysis. Second, all subjects were further divided into five equal-size groups using the quintiles of the estimated propensity score and stratified Cox regression analysis was conducted to obtain a pooled hazard ratio across the five strata to ensure the consistency among different estimates of transfusion effects on cancer recurrence or overall survival. Third, propensity score matching was done without replacement and within a tolerance limit of 0.05. The comparisons of patient variables between the paired groups were conducted as aforementioned and stratified Cox regression model by matching pairs was used to evaluate the association between blood transfusion and cancer recurrence or overall survival. All the statistical analyses were conducted with IBM SPSS Statistics, Version 23.0 (Armonk, NY: IBM Corp.). Schoenfeld’s formula for the proportional-hazards regression model was used to estimate the minimum requirement of sample size^31^. Based on the previous survey^13^, at least 208 subjects were needed to achieve a power of 0.9 given a type I error rate of 0.05 and the proportion of patients receiving blood transfusion in our study. Note that we collected near 20 times the demanded samples in this study.

## Electronic supplementary material

Supplementary Information

## Data Availability

All data generated or analysed during this study are available from the corresponding author on reasonable request.
