# Supplementary material for: The Impact of Blood Transfusion on Recurrence and Mortality Following Colorectal Cancer Resection: A Propensity Score Analysis of 4,030 Patients
Source: Sci Rep. 2018 Sep 6;8:13345. doi: 10.1038/s41598-018-31662-5 (PMC6127303; doi:10.1038/s41598-018-31662-5)
Supplement: Supplementary file 1 — Supplementary Information [file 41598_2018_31662_MOESM1_ESM.doc]

**[Supplementary Information] The Impact of Blood Transfusion on Recurrence and Mortality Following Colorectal Cancer Resection: A Propensity Score Analysis of 4,030 Patients**

Hsiang-Ling Wu ^1,2,3^, Ying-Hsuan Tai ^1,3,4,5^, Shih-Pin Lin ^1,3^, Min-Ya Chan ^1,6^, Hsiu-Hsi Chen ^7^, Kuang-Yi Chang ^1,3,*^

^1^ Department of Anesthesiology, Taipei Veterans General Hospital, Taipei, Taiwan

^2^ Department of Surgery, Taipei Veterans General Hospital, Yuli Branch, Hualien, Taiwan

^3^ School of Medicine, National Yang-Ming University, Taipei, Taiwan

^4^ Department of Anesthesiology, Shuang Ho Hospital, Taipei Medical University, New Taipei, Taiwan

^5^ Department of Anesthesiology, School of Medicine, College of Medicine, Taipei Medical University, Taipei, Taiwan.

^6^ Department of Technology Application and Human Resource Development, National Taiwan Normal University, Taipei, Taiwan

^7^ Division of Biostatistics, Graduate Institute of Epidemiology and Preventive Medicine, College of Public Health, National Taiwan University, Taipei, Taiwan

**Supplementary Table S1:** The result of logistic regression analysis for further propensity score matching

|  | **OR** | **95% C.I.** | ***p*** |
| --- | --- | --- | --- |
| **Hemoglobin concentration** | 0.421 | 0.392 – 0.452 | <.001 |
| **Platelet count** | 1.003 | 1.002 – 1.004 | <.001 |
| **INR (>1 vs.** ≤ **1)** | 1.315 | 0.316 – 5.468 | 0.707 |
| **Age** | 1.019 | 1.009 – 1.028 | <.001 |
| **Gender, M vs. F** | 1.344 | 1.083 – 1.667 | 0.007 |
| **ASA class ≥ 3** | 1.716 | 1.350 – 2.183 | <.001 |
| **Diabetes** | 1.142 | 0.903 – 1.444 | 0.268 |
| **Coronary arterial disease** | 1.271 | 0.942 – 1.715 | 0.117 |
| **Heart failure** | 1.247 | 0.864 – 1.801 | 0.239 |
| **Stroke** | 1.078 | 0.740 – 1.572 | 0.695 |
| **Chronic kidney disease** | 1.779 | 1.358 – 2.330 | <.001 |
| **Pretreatment CEA *** | 1.391 | 1.109 – 1.745 | 0.004 |
| **Right- vs. left-sided tumour** | 1.289 | 1.029 – 1.616 | 0.027 |
| **Epidural block** | 1.453 | 1.105 – 1.909 | 0.007 |
| **Anaesthesia time **** | 3.808 | 2.921 – 4.963 | <.001 |
| **Stage** |  |  | 0.046 |
| II vs. I | 0.966 | 0.704 – 1.325 | 0.830 |
| III vs. I | 1.358 | 0.931 – 1.981 | 0.112 |
| **Tumour differentiation** |  |  | 0.648 |
| Moderate vs. good | 0.878 | 0.539 – 1.430 | 0.602 |
| Poor vs. good | 1.044 | 0.550 – 1.980 | 0.896 |
| **Mucinous histology** | 1.071 | 0.652 – 1.759 | 0.786 |
| **Signet-ring histology** | 0.751 | 0.388 – 1.455 | 0.397 |
| **Lymphovascular invasion** | 0.996 | 0.737 – 1.346 | 0.980 |
| **Perineural invasion** | 1.011 | 0.678 – 1.507 | 0.956 |
| **Postoperative C/T** | 0.813 | 0.622 – 1.062 | 0.129 |
| **Postoperative R/T** | 2.021 | 0.928 – 4.403 | 0.077 |
| **Preoperative C/T ± R/T** | 1.428 | 0.982 – 2.077 | 0.062 |

OR: odds ratio; INR: international normalized ratio; M: male, F: female; ASA: American Society of Anesthesiologists; CEA: carcinoembryonic antigen; C/T: chemotherapy; R/T: radiotherapy. * On base-10 logarithmic scale; ** On base-2 logarithmic scale

**Supplementary Table S2:** Multivariable analysis between transfusion and disease-free or overall survival stratified by anemia status before matching

|  | **Anemia** | | | **No anemia** | | |
| --- | --- | --- | --- | --- | --- | --- |
|  | **HR** | **95% C.I.** | ***p*** | **HR** | **95% C.I.** | ***p*** |
| **Disease-free survival** |  |  |  |  |  |  |
| Blood transfusion | 1.526 | 1.022 – 2.277 | 0.039 | 1.434 | 1.159 – 1.774 | 0.001 |
| Pretreatment CEA* | 2.065 | 1.602 – 2.662 | <.001 | 1.754 | 1.492 – 2.062 | < 0.001 |
| Anaesthesia time** | 1.418 | 0.971 – 2.071 | 0.071 | 1.248 | 1.010 – 1.543 | 0.040 |
| Cancer stage |  |  | <.001 |  |  | <.001 |
| Stage II vs. I | 2.173 | 0.860 – 5.487 | 0.101 | 3.016 | 2.112 – 4.306 | <.001 |
| Stage III vs. I | 4.916 | 1.972 – 12.255 | 0.001 | 6.066 | 4.272 – 8.611 | <.001 |
| Signet-ring histology | 1.985 | 1.082 – 3.643 | 0.027 | 1.428 | 0.925 – 2.206 | 0.108 |
| Lymphovascular invasion | 1.459 | 0.999 – 2.131 | 0.050 | 1.330 | 1.084 – 1.632 | 0.006 |
| Perineural invasion | 1.823 | 1.126 – 2.951 | 0.015 | 1.765 | 1.388 – 2.245 | <.001 |
| Postoperative R/T | 0.852 | 0.260 – 2.794 | 0.792 | 2.695 | 1.771 – 4.101 | <.001 |
| Preoperative C/T ± R/T | 2.039 | 1.102 – 3.774 | 0.023 | 2.190 | 1.742 – 2.753 | <.001 |
| **Overall survival** |  |  |  |  |  |  |
| Blood transfusion | 2.210 | 1.252 – 3.899 | 0.006 | 2.041 | 1.560 – 2.670 | <.001 |
| Age | 1.020 | 1.001 – 1.039 | 0.043 | 1.030 | 1.018 – 1.042 | <.001 |
| Gender (M vs. F) | 1.408 | 0.941 – 2.110 | 0.096 | 1.186 | 0.923 – 1.524 | 0.182 |
| ASA class ≥ 3 | 2.669 | 1.643 – 4.336 | <.001 | 1.349 | 1.034 – 1.761 | 0.027 |
| Heart failure | 1.235 | 0.722 – 2.114 | 0.441 | 1.560 | 1.076 – 2.262 | 0.019 |
| Chronic kidney disease | 1.508 | 0.992 – 2.292 | 0.054 | 1.411 | 1.050 – 1.896 | 0.022 |
| Pretreatment CEA* | 1.484 | 1.028 – 2.140 | 0.035 | 1.638 | 1.288 – 2.084 | <.001 |
| Cancer stage |  |  | 0.018 |  |  | <.001 |
| Stage II vs. I | 1.232 | 0.596 – 2.546 | 0.573 | 1.310 | 0.904 – 1.898 | 0.154 |
| Stage III vs. I | 2.136 | 1.025 – 4.452 | 0.043 | 2.305 | 1.599 – 3.323 | <.001 |
| Lymphovascular invasion | 1.498 | 0.946 – 2.372 | 0.085 | 1.364 | 1.015 – 1.834 | 0.040 |
| Perineural invasion | 1.558 | 0.851 – 2.852 | 0.150 | 1.480 | 1.019 – 2.149 | 0.040 |
| Preoperative C/T ± R/T | 2.640 | 1.321 – 5.278 | 0.006 | 2.092 | 1.482 – 2.955 | <.001 |

HR: hazard ratio; CEA: carcinoembryonic antigen; R/T: radiotherapy; C/T: chemotherapy; M: male, F: female; ASA: American Society of Anesthesiologists.

* On base-10 logarithmic scale; ** On base-2 logarithmic scale

**Supplementary Table S3:** Forward model selection for dose response relation between transfusion and disease-free or overall survival before matching

|  | **HR** | | **95% C.I.** | | ***p*** | |  |
| --- | --- | --- | --- | --- | --- | --- | --- |
| **Disease-free survival** | |  | |  | |  | |
| Blood transfusion | |  | |  | | <.001 | |
| ≤ 4 units vs. nil | | 1.388 | | 1.135 – 1.699 | | 0.001 | |
| > 4 units vs. nil | | 1.433 | | 1.168 – 1.759 | | 0.001 | |
| Pretreatment CEA * | | 1.830 | | 1.597 – 2.097 | | <.001 | |
| Anaesthesia time ** | | 1.276 | | 1.061 – 1.533 | | 0.009 | |
| Stage | |  | |  | | <.001 | |
| II vs. I | | 2.839 | | 2.038 – 3.954 | | <.001 | |
| III vs. I | | 5.848 | | 4.221 – 8.103 | | <.001 | |
| Signet-ring histology | | 1.590 | | 1.122 – 2.254 | | 0.009 | |
| Lymphovascular invasion | | 1.371 | | 1.146 – 1.641 | | 0.001 | |
| Perineural invasion | | 1.745 | | 1.410 – 2.160 | | <.001 | |
| Preoperative C/T ± R/T | | 2.188 | | 1.768 – 2.708 | | <.001 | |
| Postoperative R/T | | 2.210 | | 1.492 – 3.273 | | <.001 | |
| **Overall survival** | |  | |  | |  | |
| Blood transfusion | |  | |  | | <.001 | |
| ≤ 4 units vs. nil | | 1.578 | | 1.200 – 2.075 | | 0.001 | |
| > 4 units vs. nil | | 2.318 | | 1.819 – 2.954 | | <.001 | |
| Age | | 1.027 | | 1.017 – 1.037 | | <.001 | |
| Gender (M vs. F) | | 1.254 | | 1.014 – 1.550 | | 0.037 | |
| ASA class ≥ 3 | | 1.598 | | 1.275 – 2.003 | | <.001 | |
| Heart failure | | 1.406 | | 1.039 – 1.903 | | 0.027 | |
| Chronic kidney disease | | 1.471 | | 1.161 – 1.864 | | 0.001 | |
| Pretreatment CEA * | | 1.533 | | 1.256 – 1.871 | | <.001 | |
| Stage | |  | |  | | <.001 | |
| II vs. I | | 1.270 | | 0.914 – 1.764 | | 0.154 | |
| III vs. I | | 2.174 | | 1.568 – 3.014 | | <.001 | |
| Mucinous histology | | 1.509 | | 1.015 – 2.245 | | 0.042 | |
| Lymphovascular invasion | | 1.420 | | 1.107 – 1.821 | | 0.006 | |
| Perineural invasion | | 1.544 | | 1.126 – 2.116 | | 0.007 | |
| Preoperative C/T ± R/T | | 2.155 | | 1.586 – 2.928 | | <.001 | |

HR: hazard ratio; CEA: carcinoembryonic antigen; C/T: chemotherapy; R/T: radiotherapy; M: male, F: female; ASA: American Society of Anesthesiologists. * On base-10 logarithmic scale; ** On base-2 logarithmic scale
